# Association between metabolic obesity phenotypes and multiple myeloma hospitalization burden: A national retrospective study

**DOI:** 10.3389/fonc.2023.1116307

**Published:** 2023-02-23

**Authors:** Yue Zhang, Xiude Fan, Chunhui Zhao, Zinuo Yuan, Yiping Cheng, Yafei Wu, Junming Han, Zhongshang Yuan, Yuanfei Zhao, Keke Lu

**Affiliations:** ^1^ Department of Endocrinology, Shandong Provincial Hospital, Shandong University, Jinan, Shandong, China; ^2^ Department of Endocrinology, Shandong Provincial Hospital Affiliated to Shandong First Medical University, Jinan, Shandong, China; ^3^ Shandong Key Laboratory of Endocrinology and Lipid Metabolism, Jinan, Shandong, China; ^4^ Shandong Institute of Endocrine and Metabolic Diseases, Jinan, Shandong, China; ^5^ Shandong Engineering Laboratory of Prevention and Control for Endocrine and Metabolic Diseases, Jinan, Shandong, China; ^6^ Department of Biostatistics, School of Public Health, Shandong University, Jinan, Shandong, China; ^7^ Beijing Institute of Heart, Lung and Blood, Beijing Anzhen Hospital, Capital Medical University, Beijing, China; ^8^ Beijing Advanced Innovation Center for Big Data-based Precision Medicine, Capital Medical University, Beijing, China

**Keywords:** MM, multiple myeloma, metabolic disorders, obesity phenotypes, hospitalization burden, readmission

## Abstract

**Background & purpose:**

Obesity and metabolic disorders were associated with increased risk of MM, a disease characterized by high risk of relapsing and require frequent hospitalizations. In this study, we conducted a retrospective cohort study to explore the association of metabolic obesity phenotypes with the readmission risk of MM.

**Patients & methods:**

We analyzed 34,852 patients diagnosed with MM from the Nationwide Readmissions Database (NRD), a nationally representative database from US. Hospitalization diagnosis of patients were obtained using ICD-10 diagnosis codes. According to obesity and metabolic status, the population was divided into four phenotypes: metabolically healthy non-obese (MHNO), metabolically unhealthy non-obese (MUNO), metabolically healthy obese (MHO), and metabolically unhealthy obese (MUO). The patients with different phenotypes were observed for hospital readmission at days 30-day, 60-day, 90-day and 180-day. Multivariate cox regression model was used to estimate the relationship between obesity metabolic phenotypes and readmissions risk.

**Results:**

There were 5,400 (15.5%), 7,255 (22.4%), 8,025 (27.0%) and 7,839 (35.6%) unplanned readmissions within 30-day, 60-day, 90-day and 180-day follow-up, respectively. For 90-day and 180-day follow-up, compared with patients with the MHNO phenotype, those with metabolic unhealthy phenotypes MUNO (90-day: P = 0.004; 180-day: P = < 0.001) and MUO (90-day: P = 0.049; 180-day: P = 0.004) showed higher risk of readmission, while patients with only obesity phenotypes MHO (90-day: P = 0.170; 180-day: P = 0.090) experienced no higher risk. However, similar associations were not observed for 30-day and 60-day. Further analysis in 90-day follow-up revealed that, readmission risk elevated with the increase of the combined factor numbers, with aHR of 1.068 (CI: 1.002-1.137, P = 0.043, with one metabolic risk factor), 1.109 (CI: 1.038-1.184, P = 0.002, with two metabolic risk factors) and 1.125 (95% CI: 1.04-1.216, P = 0.003, with three metabolic risk factors), respectively.

**Conclusion:**

Metabolic disorders, rather than obesity, were independently associated with higher readmission risk in patients with MM, whereas the risk elevated with the increase of the number of combined metabolic factors. However, the effect of metabolic disorders on MM readmission seems to be time-dependent. For MM patient combined with metabolic disorders, more attention should be paid to advance directives to reduce readmission rate and hospitalization burden.

## Introduction

Multiple myeloma (MM), the second most common hematological malignancy, accounts for 1% of all tumor diseases (1). Although the treatment of MM makes significant progress, MM remains a severe and incurable disease, and most patients relapse and require frequent hospitalizations (2, 3). Hospital readmission is a indicator of medical resource utilization and nursing quality evaluation, and also an important predictor of disease outcomes, such as increased morbidity, raised mortality, and the loss of functional independence (4, 5). The annual cost of readmissions within 30 days after discharge accounts for more than $17 billion of avoidable Medicare expenses in the United States (6). It is foreseeable that repeat hospitalization of MM patients will be a significant burden on the healthcare system and patients. Therefore, there is an urgent need to identify risk factors of readmission to reduce this significant and continuous burden.

Obesity, an urgent and growing global public health threat, is a significant risk factor for chronic diseases such as cardiovascular disease (CVD), diabetes, and certain types of cancer (7, 8). Although evidence suggests that obesity is associated with higher incidence and mortality in MM (9), the effect of obesity on MM is still controversial (10, 11). Studies suggested that metabolically healthy obese (MHO) individuals are at lower risk of CVD, cancers and mortality than those metabolically unhealthy obese (MUO) (12, 13). Similarly, normal-weight individuals with unhealthy metabolic characteristics (metabolically unhealthy non-obese [MUNO]) have increased risk of CVD than those with metabolically healthy status (metabolically healthy non-obese [MHNO]) (13). These differences implied that in addition to obesity, taking into account the coexistence of metabolic abnormalities can more effective in identifying risk factors for obesity-related diseases (9–11). Given the conflicting evidence on the relationship between obesity and MM, it seems to be more appropriate to consider the obesity and metabolic jointly to study the risk factors for MM.

Therefore, to accurately and systematically explore the potential modifiable risk factors for MM, we conducted a retrospective cohort study using the Nationwide Readmissions Database (NRD), a large contemporary nationwide database from the United States. We evaluated the differences in readmission risk among patients with different combinations of obesity and metabolic status to identify risk factor of readmission, and provide a clinical reference for optimizing patient care and minimizing the medical burden.

## Patients and methods

### Data sources

We conducted a retrospective cohort study using 2018 data from the NRD database (https://hcup-us.ahrq.gov/db/nation/nrd/nrddbdocumentation.jsp). The NRD is a longitudinal database developed and maintained by the Agency for Healthcare Research and Quality from the US. The NRD 2018 contains data from 28 states including clinical and non-clinical information at the hospital and patient levels, representing 60 percent of total population and 58.7 percent of hospitalizations in the United States (14). Rehabilitation and long-term care hospitals are not included. Based on patient linkage numbers, the NRD can track individuals across hospitals within a state. Because of the de-identified nature of the data, this study was determined to be exempt from ethics board review by the local ethics committee, Biomedical Research Ethic Committee of Shandong Provincial Hospital.

### Population

**Figure 1:**
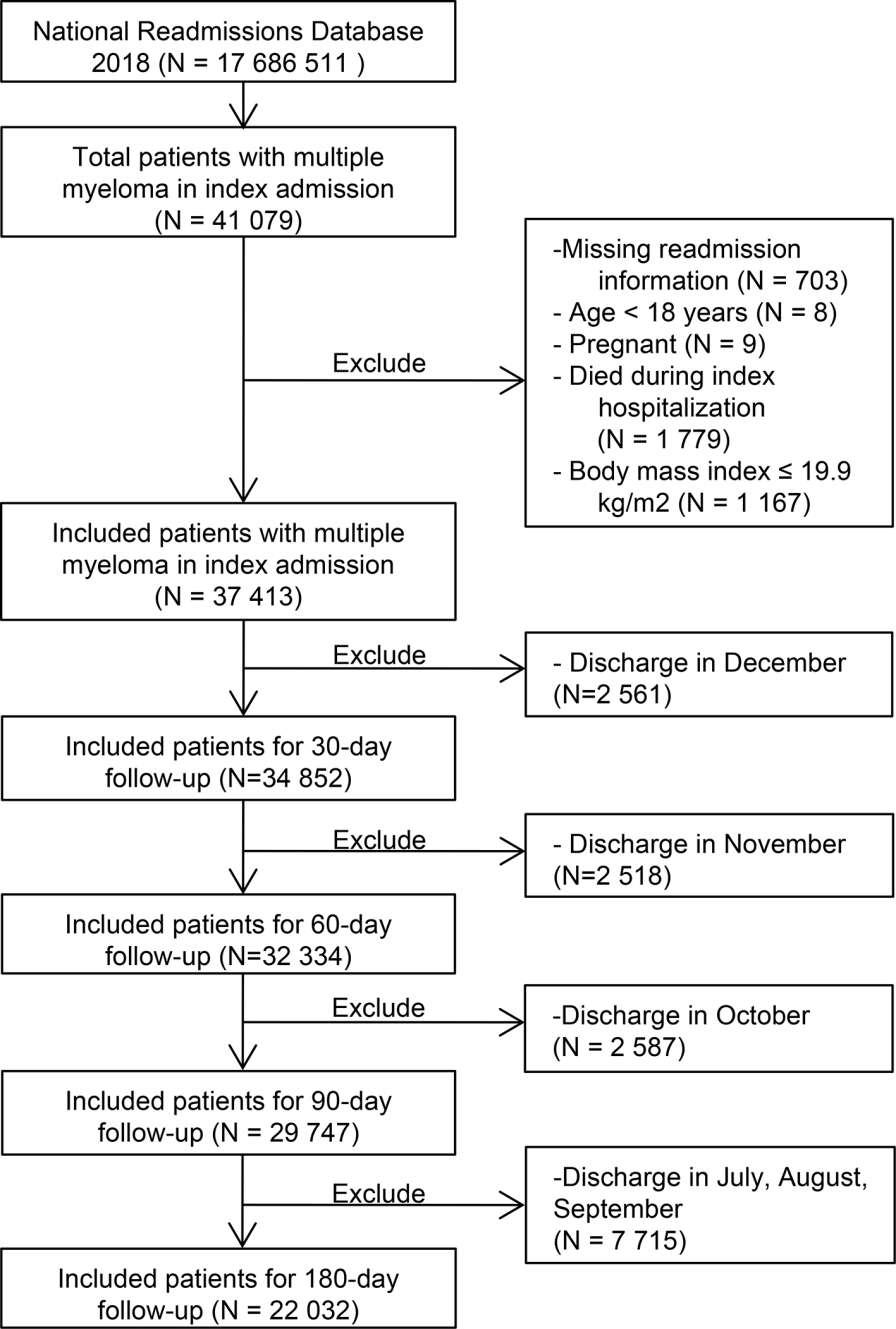
Flow chart of data screening.

We used the International Classification of Diseases-Tenth Revision-Clinical Modification (ICD-10-CM) codes (c90.x based) to identify patients with the first 30 diagnoses including MM on the index discharge. Due to data limitations, we did not consider the clinical stage, karyotype, oncogenic mutations and treatment strategies of MM patients in this study. The codes used to diagnose disease were listed in **Supplementary Table 1**. Patients with missing important hospitalization information, aged under 18 years old, pregnant, died during the index hospitalization, and low body weight [body mass index (BMI) ≤ 19.9 kg/m^2^] were excluded. Because the NRD cannot track admissions for the following year, we excluded those discharged in December for the 30-day follow-up study; for 60-day, we excluded those who were discharged in November and December; for 90-day, we excluded those who were discharged from October to December, and for the 180-day, we excluded those who were discharged from July to December. Detailed inclusion and exclusion criteria were shown in **Figure 1**.

### Patient characteristics

For each patient, demographic variables and hospitalization characteristics were collected, which included age, gender, primary payer status (Medicare, Medicaid, private insurance, self-pay, free, and other types), median household income by ZIP code, discharge disposition, location, length of stay (LOS), total charges and comorbidities. Detailed information was provided in **Table 1**.

**Table 1:** Baseline clinical and demographic characteristics of index hospitalizations for patients.

Variables	Total	MHNO	MUNO	MHO	MUO	P value*
No. of cases	34852	17305 (49.7%)	12829 (36.8%)	2130 (6.1%)	2588 (7.4%)	
**Age (years) (mean (SD))**	69.8 (11.4)	68.2 (12.1)^a^	73.3 (9.8)^b^	64.4 (11.6)^c^	68.5 (9.9)^a^	<0.001
**Age ≥ 65**	24178 (69.4%)	10908 (63.0%)^a^	10475 (81.7%)^b^	1069 (50.2%)^c^	1726 (66.7%)^d^	<0.001
**Male**	19262 (55.3%)	9388 (54.3%)a	7519 (58.6%)b	1064 (50.0%)c	1291 (49.9%)c	<0.001
**Primary payer**						<0.001
Medicare	24389 (70.0%)	11130 (64.3%)^a^	10228 (79.7%)^b^	1202 (56.4%)^c^	1829 (70.7%)^d^	
Medicaid	2078 (6.0%)	1177 (6.8%)^a^	535 (4.2%)^b^	205 (9.6%)^c^	161 (6.2%)^a^	
Private insurance	7281 (20.9%)	4376 (25.3%)^a^	1725 (13.4%)^b^	646 (30.3%)^c^	534 (20.6%)^d^	
Self-pay	332 (1.0%)	198 (1.1%)^a^	102 (0.8%)^b^	17 (0.8%)^a, b^	15 (0.6%)^a, b^	
Free	66 (0.2%)	45 (0.3%)^a^	11 (0.1%)^a^	5 (0.2%)^a, b^	5 (0.2%)^a, b^	
Other	706 (2.0%)	379 (2.2%)^a^	228 (1.8%)^a^	55 (2.6%)^a^	44 (1.7%)^a^	
**Median household income by ZIP code**						<0.001
0-25th	8406 (24.1%)	3929 (22.7%)^a^	3262 (25.4%)^b, c^	505 (23.7%)^a, c^	710 (27.4%)^b^	
26-50th	8985 (25.8%)	4390 (25.4%)^a^	3294 (25.7%)^a, b^	581 (27.3%)^a, b^	720 (27.8%)^b^	
51-75th	8870 (25.5%)	4456 (25.7%)^a^	3218 (25.1%)^a^	572 (26.9%)^a^	624 (24.1%)^a^	
76-100th	8591 (24.6%)	4530 (26.2%)^a^	3055 (23.8%)^b^	472 (22.2%)^b, c^	534 (20.6%)^c^	
**Disposition**						<0.001
Routine	20250 (58.1%)	10840 (62.6%)^a^	6843 (53.3%)^b^	1228 (57.7%)^c^	1339 (51.7%)^d^	
Short-term Hospital	398 (1.1%)	190 (1.1%)^a^	161 (1.3%)^a^	27 (1.3%)^a^	20 (0.8%)^a^	
Skilled Nursing/ Other Facility	6179 (17.7%)	2717 (15.7%)^a^	2559 (19.9%)^b^	387 (18.2%)^b^	516 (19.9%)^b^	
Home Health Care	7787 (22.3%)	3427 (19.8%)^a^	3189 (24.9%)^b, c^	473 (22.2%)^a, c^	698 (27.0%)^b^	
Against Medical Advice	225 (0.6%)	125 (0.7%)^a^	71 (0.6%)^a^	15 (0.7%)^a^	14 (0.5%)^a^	
Discharge alive, destination unknown	13 (0.0%)	6 (0.0%)^a^	6 (0.0%)^a^	0 (0.0%)^a^	1 (0.0%)^a^	
**Location**						0.007
Large central counties	10033 (28.8%)	4871 (28.1%)^a^	3847 (30.0%)^b^	571 (26.8%)^a^	744 (28.7%)^a, b^	
Large fringe counties	9795 (28.1%)	4813 (27.8%)^a^	3652 (28.5%)^a^	594 (27.9%)^a^	736 (28.4%)^a^	
Medium metro counties	7309 (21.0%)	3697 (21.4%)^a^	2606 (20.3%)^a^	478 (22.4%)^a^	528 (20.4%)^a^	
Small metro counties	3269 (9.4%)	1652 (9.5%)^a^	1144 (8.9%)^a^	218 (10.2%)^a^	255 (9.9%)^a^	
Micropolitan counties	2489 (7.1%)	1250 (7.2%)^a^	895 (7.0%)^a^	159 (7.5%)^a^	185 (7.1%)^a^	
Not metro/ micropolitan counties	1957 (5.6%)	1022 (5.9%)^a^	685 (5.3%)^a^	110 (5.2%)^a^	140 (5.4%)^a^	
**LOS (days) (mean (SD))**	7.4 (8.9)	7.4 (8.9)^a^	6.9 (8.4)^b^	9.5 (11.6)^c^	8.1 (8.4)^d^	<0.001
**LOS > 7 days**	10861 (31.2%)	5376 (31.1%)^a^	3696 (28.8%)^b^	860 (40.4%)^c^	929 (35.9%)^d^	<0.001
**Total Charges ($) (mean (SD))**	87260.1 (121839.4)	88067.5 (126436.0)^a^	80442.4 (109012.6)^b^	112913.9 (146002.6)^c^	94544.4 (125521.9)^b^	<0.001
Comorbidities						
Metabolically unhealthy	15417 (44.2%)	0 (0.0%)^a^	12829 (100.0%)^b^	0 (0.0%)^a^	2588 (100.0%)^b^	<0.001
Obesity	4718 (13.5%)	0 (0.0%)^a^	0 (0.0%)^a^	2130 (100.0%)^b^	2588 (100.0%)^b^	<0.001
Hyperglycemia	10108 (29.0%)	846 (4.9%)^a^	7272 (56.7%)^b^	168 (7.9%)^c^	1822 (70.4%)^d^	<0.001
Dyslipidemia	13126 (37.7%)	1343 (7.8%)^a^	9801 (76.4%)^b^	150 (7.0%)^a^	1832 (70.8%)^c^	<0.001
Hypertension	24418 (70.1%)	8306 (48.0%)^a^	12417 (96.8%)^b^	1198 (56.2%)^c^	2497 (96.5%)^d^	<0.001
Heart failure	7826 (22.5%)	2661 (15.4%)^a^	3880 (30.2%)^b^	371 (17.4%)^a^	914 (35.3%)^c^	<0.001
Renal failure	17244 (49.5%)	7233 (41.8%)^a^	7507 (58.5%)^b^	956 (44.9%)^c^	1548 (59.8%)^d^	<0.001
Coronary heart disease	6752 (19.4%)	1845 (10.7%)^a^	4009 (31.2%)^b^	206 (9.7%)^a^	692 (26.7%)^c^	<0.001
Neoplastic anemia	2938 (8.4%)	1470 (8.5%)^a^	1072 (8.4%)^a^	185 (8.7%)^a^	211 (8.2%)^a^	0.891
Neutropenia	2034 (5.8%)	1295 (7.5%)^a^	490 (3.8%)^b^	159 (7.5%)^a^	90 (3.5%)^b^	<0.001
Depression	4058 (11.6%)	1823 (10.5%)^a^	1574 (12.3%)^b^	281 (13.2%)^b, c^	380 (14.7%)^c^	<0.001

LOS, length of stay; MHNO, metabolically healthy nonobese; MUNO, metabolically unhealthy nonobese; MHO, metabolically healthy obese; MUO, metabolically unhealthy obese.

Values are No. (%) or as mean (SD) indicated. *P values were for the χ2 test or the ANOVA across the four categories of obesity phenotypes. The small letters (e.g., a, b, c, d) in this table refer to comparisons between groups. There is no statistical difference between groups with the same small letters.

### Definition

The readmission was defined as unplanned readmission to the hospital due to any diagnosis within 30 days, 60 days, 90 days, and 180 days from index discharge. If there were more than one readmission, only the first readmission was counted. Over-weight and obesity was defined as BMI ≥ 25 kg/m^2^ (15, 16). There was still lack of consensus on the definition of metabolic health status. However, many studies defined metabolic unhealthy status as presence of ≥2 metabolic risk factors (17, 18). According to the Adult Treatment Panel III (ATP-III) criteria and the International Diabetes Federation (IDF) consensus (19, 20), we defined metabolic risk factors including (1) hypertension: primary hypertension or secondary hypertension or undiagnosed elevated blood pressure; (2) dyslipidemia: high serum triglyceride (TGs) levels or high high-density lipoprotein (HDL)-cholesterol levels, etc.; (3) hyperglycemia: pre-diabetes or diabetes mellitus or other specific diabetes. Abdominal obesity was not included in the models because of the collinearity of waist circumference and BMI. Metabolically unhealthy status was defined as with two or more of the above metabolic risk factors. The codes used were listed in **Supplementary Table 1**. Based on the obesity and metabolic status, individuals were classified into four different phenotypes; (1) metabolically healthy non-obesity (MHNO); (2) metabolically unhealthy non-obesity (MUNO); (3) metabolically healthy obesity (MHO); and (4) metabolically unhealthy obesity (MUO). For 90-day analysis, based on combined metabolic risk factor type only, individuals were further divided into: (1) no metabolic risk factor; (2) only with hyperglycemia; (3) only with dyslipidemia; (4) only with hypertension. In addition, based on the number of combined metabolic risk factors, individuals were further divided into: (1) no metabolic risk factor; (2) one metabolic risk factor; (3) two metabolic risk factors; (4) three metabolic risk factors.

### Outcomes

The primary outcome of the study was all-cause unplanned readmissions of different metabolic obesity phenotypes for four follow-up days among patients with MM. The secondary outcomes were the LOS, mean total hospitalization charges, and readmission mortality during the readmission.

### Statistical analysis

We used descriptive statistics to compare the demographic and admission characteristics of patients in different metabolic obesity phenotypes. The Chi-square test was used to analyze categorical variables, and the ANOVA was used to analyze continuous variables. Categorical variables were presented as counts with percentages. Continuous variables were presented as means and standard deviation (SD). We estimated the unadjusted hazard ratio (HR) with the univariate Cox proportional hazards model. In addition, the multivariate Cox regression model was used to analyze adjusted HR (aHR) value including the potential confounding factors. Based on the associations found from the literature and univariate analysis, we adjusted factors such as age, gender, elective versus non-elective admission, primary payer, disposition of patient, resident, length of stay, total charges, emergency record, same day events, patient location, antineoplastic chemotherapy, and stem cells transplant status. All calculated P-values were two-sided, and the threshold for significance was set at P < 0.05. All statistical analyses were performed by SPSS software (version 26.0; SPSS, Chicago, IL).

## Results

### Patient baseline characteristics

From 17,686,511 discharge records in NRD 2018, we included 34,852 patients diagnosed with MM at index discharge in the cohort study (**Figure 1**). For 30-day, 60-day, 90-day and 180-day follow-ups, we analyzed 34,852, 32,334, 29,747 and 22,032 participants, respectively (**Figure 1**). **Table 1** provided the demographic characteristics and common comorbidities of patients with different metabolic obesity phenotypes.

The mean age of the population was 69.8 years old, and the elderly aged 65 or above accounted for 69.4% (**Table 1**). The majority of patients were male (55.3%) and used privately insured (70.0%). There were 4718 (13.5%) patients with obesity and 15417 (44.2%) patients with metabolically unhealthy status.

The MHNO group was the largest of the four phenotypes, and the MHO had the longest mean LOS (9.5 days) and highest mean total charges ($112913.9). Compared with MHNO and MHO groups, MUNO and MUO groups had more elder patients (>65 years) and higher prevalence of heart failure, renal failure, and coronary heart disease. MUNO, MHO, and MUO groups had higher prevalence of depression than the MHNO group (**Table 1**).

### Readmission risk

**Figure 2:**
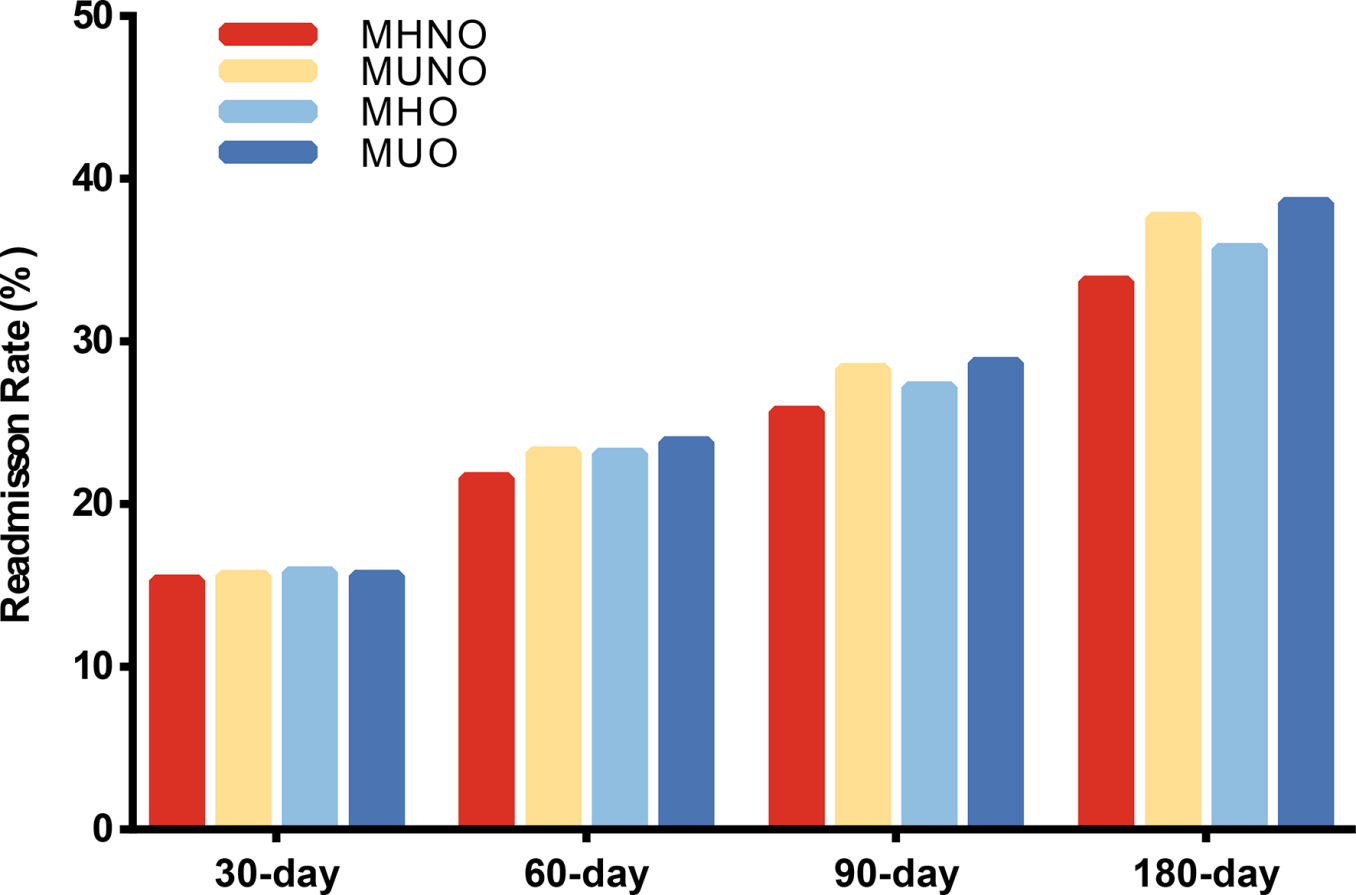
Readmission rate among different metabolic obesity phenotypes in four follow-up days. MHNO, metabolically healthy non-obese; MUNO, metabolically unhealthy non-obese; MHO, metabolically healthy obese; MUO, metabolically unhealthy obese.

For 30-day, 60-day, 90-day and 180-day follow-up, we observed that 5,400 (15.5%), 7,255 (22.4%), 8,025 (27.0%) and 7,839 (35.6%) patients experienced unplanned readmissions (**Supplementary Table 2**), respectively. **Figure 2** showed the readmission rate among different metabolic obesity phenotypes in the four cohorts. The MUNO and MUO groups had higher readmission rate than MHNO and MHO groups in 60-day, 90-day, and 180-day studies, respectively (**Figure 2**).

**Figure 3:**
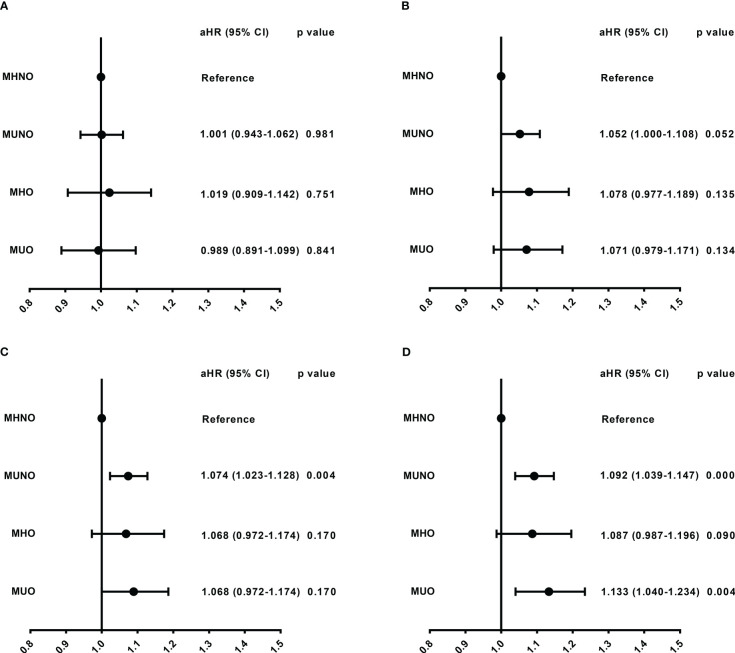
Readmission risk among different metabolic obesity phenotypes in four follow-up days. **(A)** Readmission risk in 30-day. **(B)** Readmission risk in 60-day. **(C)** Readmission risk in 90-day. **(D)** Readmission risk in 180-day. The model was adjusted for age, sex, elective admission, primary payer, disposition of patient, resident, length of stay, total charges, emergency record, same day events, patient location, antineoplastic chemotherapy, and stem cells transplant status. aHR, adjusted hazard ratio; CI, confidence interval; MHNO, metabolically healthy non-obese; MUNO, metabolically unhealthy non-obese; MHO, metabolically healthy obese; MUO, metabolically unhealthy obese.

In the 30-day and 60-day research, we observed that there were no differences in readmission risk among different metabolic obesity phenotypes (**Figures 3A, B**, **Supplementary Table 2**). In the 90-day and 180-day research, patients in the metabolically unhealthy groups (MUNO and MUO) had a higher readmission rate than those in the metabolically healthy group (MHNO and MHO), respectively (**Supplementary Table 2**). In 90-day analysis, patients in the MUNO group [aHR = 1.07 (1.023-1.128), P = 0.004] and the MUO group [aHR = 1.089 (1.000-1.186), P = 0.049] had higher readmission risk than those in the MHNO group (**Figure 3C**, **Supplementary Table 2**). In 180-day analysis, we also observed higher risk of readmission in the MUNO group [aHR = 1.092 (1.039-1.147), P = < 0.001] and the MUO group [aHR = 1.133 (1.040-1.234), P = 0.004] than MHNO (**Figure 3D**, **Supplementary Table 2**), respectively.

The 90-day data was then processed for further analysis. When stratified by age, higher risk in the MUNO group [aHR = 1.063 (1.005-1.125), P = 0.032] and the MUO group [aHR = 1.12 (1.009-1.242), P = 0.033] than in the MHNO was only observed in the elderly population aged 65 or older (**Supplementary Table 3**). As shown in subgroups based on metabolic risk factor type (**Table 2**), patients with hyperglycemia only [HR = 1.193 (1.044-1.364), P = 0.01] and hypertension only [HR = 1.091 (1.022-1.164), P = 0.009] had increased risk of readmission compared with those without metabolic risk factors. However, similar result was not observed after correction for confounding factors. In subgroups based on the number of metabolic risk factors, we found that patients with metabolic risk factors had higher readmission risk than those without metabolic risk factors. Furthermore, the risk elevated with the increase of the number of combined factors (**Table 2**), with aHR of 1.068 (P = 0.043, combined with one metabolic risk factor), 1.109 (P = 0.002, combined with two metabolic risk factors), and 1.125 (P = 0.003, combined with three metabolic risk factors).

**Table 2:** Readmission risk of different metabolic status groups in 90-day follow-up.

	Total Number	Number of Readmissions	HR (95% CI)	P value	aHR* (95% CI)	P value
Metabolic obesity phenotypes						
MHNO	14810	3804 (25.7%)	Reference		Reference	
MUNO	10930	3098 (28.3%)	1.113 (1.061-1.167)	< 0.001	1.074 (1.023-1.128)	0.004
MHO	1829	497 (27.2%)	1.063 (0.968-1.168)	0.198	1.068 (0.972-1.174)	0.170
MUO	2178	626 (28.7%)	1.131 (1.039-1.231)	0.004	1.089 (1.000-1.186)	0.049
Metabolic risk factor type						
No Metabolic Risk Factor	6346	1560 (24.6%)	Reference		Reference	
Only With Hyperglycemia	866	249 (28.8%)	1.193(1.044-1.364)	0.010	1.141 (0.997-1.305)	0.056
Only With Dyslipidemia	1275	328 (25.7%)	1.054(0.936-1.188)	0.383	1.061 (0.940-1.196)	0.339
Only With Hypertension	8152	2164 (26.5%)	1.091(1.022-1.164)	0.009	1.056 (0.988-1.129)	0.109
Metabolic risk factor numbers						
No Metabolic Risk Factor	6346	1560 (24.6%)	Reference		Reference	
One Metabolic Risk Factor	10293	2741 (26.6%)	1.095 (1.029-1.166)	0.004	1.068 (1.002-1.137)	0.043
Two Metabolic Risk Factors	9012	2540 (28.2%)	1.162 (1.091-1.238)	< 0.001	1.109 (1.038-1.184)	0.002
Three Metabolic Risk Factors	4096	1184 (28.9%)	1.198 (1.110-1.292)	< 0.001	1.125 (1.040-1.216)	0.003

aHR, adjusted hazard ratio; CI, confidence interval; HR, hazard ratio; MHNO, metabolically healthy nonobese; MUNO, metabolically unhealthy nonobese; MHO, metabolically healthy obese; MUO, metabolically unhealthy obese.

*Adjusted COX regression: adjusted for age, sex, elective versus non-elective admission, primary payer, disposition of patient, resident, length of stay, total charges, emergency record, same day events, patient location, antineoplastic chemotherapy, stem cells transplant status, obesity.

### Death and healthcare during readmission

Of the patients discharged from their index hospitalization and followed for 30-days, 60-days, 90-days, and 180-days, 5400 (15.5%), 7255 (22.4%), 8025 (27.0%), 7839 (35.6%) were readmitted, respectively. During the readmission, the MUO group were more likely to have LOS over 7 days than those in the MHNO group at 30-days (36.3% vs. 28.5%, p < 0.05), 60-days (35.1% vs. 28.1%), p < 0.05), and 90-days (32.9% vs. 27.3%, p < 0.05) (**Table 3**). At 90-day readmission, patients in the MHO group had higher total charges than the MUNO group ($ 88752.3 vs. $ 73876.1, p < 0.05; **Table 3**). However, no significant difference in mortality was found among the four metabolic obesity phenotypes.

**Table 3:** Death and healthcare during readmission.

Variables	Total	MHNO	MUNO	MHO	MUO	P value*
30-day						
total charge ($) (mean (SD))	81234.7 (125096.4)	81443.5 (119524.0)	78280.3 (127669.8)	97277.6 (170249.6)	81190.3 (100843.0)	0.084
LOS>7 days	1604 (29.7%)	756 (28.5%)^a^	592 (29.5%)^a^	109 (32.4%)^a, b^	147 (36.3%)^b^	0.009
Died	415 (7.7%)	187 (7.0%)	163 (8.1%)	33 (9.8%)	32 (7.9%)	0.236
60-day						
total charge ($) (mean (SD))	79189.5 (119116.5)	79462.1 (115793.5)^a^	76875.6 (120066.0)^a^	90473.4 (155932.8)^a^	79680.9 (98411.9)^a^	0.002
LOS>7 days	2100 (28.9%)	976 (28.1%)^a^	784 (28.5%)^a^	141 (30.9%)^a, b^	199 (35.1%)^b^	0.005
Died	494 (6.8%)	233 (6.7%)	184 (6.7%)	38 (8.3%)	39 (6.9%)	0.613
90-day						
total charge ($) (mean (SD))	76972.9 (113977.2)	78094.8 (112106.4)^a,b^	73876.1 (113771.1)^a^	88752.3 (148659.1)^b^	76129.4 (92002.0)^a,b^	0.046
LOS>7 days	2232 (27.8%)	1 040 (27.3%)^a^	839 (27.1%)^a^	147 (29.6%)^a, b^	206 (32.9%)^b^	0.018
Died	529 (6.6%)	253 (6.7%)	197 (6.4%)	41 (8.2%)	38 (6.1%)	0.425
180-day						
total charge ($) (mean (SD))	75536.0 (109226.8)	76448.0 (108947.0)	72910.9 (107397.7)	86417.3 (136745.0)	74623.8 (94644.7)	0.079
LOS>7 days	2116 (27.0%)	990 (26.7%)^a^	786 (25.8%)^a^	150 (31.5%)^a^	190 (30.8%)^a^	0.008
Died	458 (5.8%)	212 (5.7%)	181 (5.9%)	32 (6.7%)	33 (5.4%)	0.781

MHNO, metabolically healthy nonobese; MUNO, metabolically unhealthy nonobese; MHO, metabolically healthy obese; MUO, metabolically unhealthy obese; USD, USA dollar; LOS, length of stay.

*P values were for the χ2 test or the ANOVA across the four categories of obesity phenotypes. The small letters (e.g., a, b) in this table refer to comparisons between groups. There is no statistical difference between groups with the same small letters.

## Discussion

With the increasing prevalence of MM in elderly patients, it is important to focus on its risk factors to identify high-risk patients and reduce the burden of disease. In this retrospective cohort study based on a representative NRD database, we analyzed 34,852 patients diagnosed with MM from the National Readmission Database (NRD) in the United States. According to obesity and metabolic status, the population was divided into four phenotypes: metabolically healthy non-obese (MHNO), metabolically unhealthy non-obese (MUNO), metabolically healthy obese (MHO), and metabolically unhealthy obese (MUO). The patients with different phenotypes were observed for hospital readmission at days 30-day, 60-day, 90-day and 180-day. We found that the metabolically unhealthy (MUNO and MUO) individuals had higher risk of 90-day and 180-day readmission than MHNO individuals. In the age subgroup analysis of 90-day, we observed similar results only in the older group (≥ 65 years of age). Moreover, further analysis found that the risk of readmission increased significantly as the number of metabolic risk factors increased. Analysis of hospitalization characteristics of readmission patients showed that patients with metabolically unhealthy obesity (MUO) were at a higher risk of long-term hospitalization than patients in other groups.

MM is a hematological malignancy with genetic abnormalities. Patients with MM had higher incidence of some modifiable risk factors, such as obesity, hyperglycemia, dyslipidemia, and hypertension (21–23). This is more likely due to the metabolic dysregulation of MM. To ensure energy demands for rapid cell proliferation and tumor growth, myeloma cells reprogram the metabolic pathways, involving metabolic reorganization of glycolysis and oxidative phosphorylation, abnormal fatty acid metabolism, and chronic inflammation (24, 25). Overweight and obesity are associated with increased morbidity and mortality risk of MM through inflammatory cytokines, leptin, insulin, and insulin-like growth factor levels (26). Previous research found that obesity could increase the number and size of bone marrow adipocytes to obtain energy and induce the overexpression of protumor cytokines (27). For MM patients with diabetes, tumor cells can evade apoptosis by insulin resistance, hyperinsulinemia, and overproduction of insulin-like growth factor 1 (25). Dyslipidemia in MM has also been widely reported. The prevailing view is that the binding of paraproteins to serum lipoproteins and related tissue may result in reduced lipoproteins clearance (21, 28). However, some studies have also pointed out that myeloma cells are dependent upon exogenous cholesterol for survival, such as low density lipoprotein that is an important antiapoptotic drug and may prevent myeloma cell apoptosis and promote myeloma cell survival (29). In addition, the higher incidence of hypertension is related to the increased cardiovascular complications in MM patients (23).

However, previous studies did not compare the effects of obesity and metabolic disorders on MM. Our analyses indicated that metabolic status rather than obesity was the determinant risk of MM readmissions. This seems to confirm that metabolic disorders have a stronger effect on MM than obesity in some respects. We found that the prevalence of cardiovascular complications was significantly higher in the metabolically unhealthy (MUNO and MUO) groups than in the metabolically healthy (MHNO and MHO) groups, which suggests that the readmission risk of metabolically unhealthy patients may be related to a higher burden of comorbidities. This was consistent with the conclusions of some previous studies (13, 30–32). In a prospective cohort study with 30 years follow-up, metabolically unhealthy individuals are at higher cardiovascular disease risk across all BMI categories, and the transition from metabolically healthy status to unhealthy phenotypes is also associated with increased cardiovascular disease risk (33). MUO is more likely to show insulin resistance, adverse fat distribution, higher inflammation markers, and adipose tissue dysfunction than MHO (13, 34). In fact, these factors are associated with higher risk of atherosclerosis and cardiovascular disease (35, 36). Furthermore, as an elderly disease, MM is more likely to be affected by severe comorbidities. Additionally, metabolic disorders may affect the clinical manifestations of MM in terms of the drug efficacy and tumor cell activity (25). Metabolically unhealthy patients may face a lower intensity of treatment (21, 34) and poorly executed care (25), which will affect the treatment efficacy of the disease.

Although there has been substantial evidence to show that obesity is the only modifiable risk factor for MM (26, 37), other studies have observed different phenomena. Increased BMI was not significantly associated with adverse outcomes in MM patients (10, 38) and was even associated with lower morbidity and longer overall survival (11). As suggested in recent literature, the influence of BMI on MM may depend on different disease stages (26). For normal individuals or patients in the early stages of the disease, elevated BMI was associated with higher mortality and disease progression, while for MM patients in a transplant or relapsing state, a higher BMI meant patients could tolerate better treatment and experience less disease-related weight loss (26). In summary, the role of obesity in MM is complex. However, previous studies did not consider the correlation between obesity and metabolic disorders. Our study found that the MM-related readmission risk, as a result of disease progression or unexpected complications, was affected by metabolic disorders, which may imply that modifiable metabolic risk factors play a more significant role than obesity in the short-term disease progression of MM patients.

As mentioned above, all metabolic risk factors can induce adverse outcomes in MM patients through multiple pathways and mechanisms. Our analysis demonstrated the cumulative effect of metabolic risk factors numbers on the risk of MM readmission, which was supported by previous studies (39). This result is worrying for MM patients with multiple metabolic risk factors, especially for the elderly patients. Interestingly, we also observed that the association between metabolically unhealthy status and readmission risk appeared in 90-day and 180-day studies, rather than in 30-day and 60-day studies. Disease progression within 60 days after treatment is commonly referred to as refractory MM (40), which is often related to cytogenetic risk (40, 41). This might indicate that, unlike the short-term influence of refractory mechanisms, metabolic disorders influence the longer-term development of MM.

Although few studies directly explore the association between metabolic disorders and obesity in MM, based on our findings, we speculate that the higher risk of readmission in metabolically unhealthy patients with MM is related, at least in part, to the above mechanisms. From our study, we have reason to believe that metabolic disorders have a greater impact on MM readmissions than obesity. We found that patients with metabolically unhealthy status had a higher readmission rate and readmission risk than those with metabolically healthy status. Meanwhile, patients with metabolic abnormalities and obesity may have a longer time in re-hospitalization stay. Through targeted interventions, such as diet, exercise and drug treatment, metabolic risk factors and quantity can be controlled to reduce the occurrence of adverse disease outcomes reduce the disease burden of MM. Readmission is considered to be a result of disease progression or unexpected complications on initial admission. Although our study may help to identify people who have a high risk of readmission, it is not clear whether the readmission of metabolically unhealthy patients is due to progression of the disease itself or related complications. Therefore, further investigations are necessary to explore the mechanisms of the effect of metabolic disorders on the development of MM.

Although a large sample size of patients was used for follow-up, our study had many other limitations. First, all diseases were diagnosed based on the ICD-10 codes, and we could not verify the accuracy of disease diagnoses in our study. Secondly, obesity defined by BMI did not consider the effect of fat distribution on the disease, which had implications for MM outcomes (15). Moreover, due to data limitations, the stage, karyotype, oncogenic mutations and treatment regimen information of MM patients were not available, which could have influenced the results. To reduce these effects, we corrected for patient antitumor chemotherapy and stem cell transplant status in the analysis, which were the primary treatment for MM. Although we did not perform a subgroup analysis based on MM stage and treatment type due to the limitations of the database, the results of this study have good representativeness and reliability among MM patients based on the large patient population in the NRD database. Further studies in more detailed cohorts are required to validate the relationship of obesity and metabolism to MM specific disease characteristics. In addition, the analyzed data were from NRD 2018, before the coronavirus disease 2019 (COVID-19) epidemic, and we were unable to assess the NRD data during the pandemic. Therefore, our analysis could not take into account the effect of COVID-19 on the results. Recent studies have shown that COVID-19 increased rehospitalizations and mortality rates in patients and had adverse effects on cancer patients, including MM (42–44). However, obesity and metabolic risk factors contribute to COVID-19 infection and adverse outcomes and increase the risk of hospitalization (45, 46). Thus, we speculate that during the pandemic, COVID-19, obesity and metabolic risk factors could increase the risk of readmission and adverse outcomes in MM patients jointly. Future clinical practice should focus on patients with obesity and metabolic abnormalities, control the occurrence of metabolic abnormalities and the number of metabolic risk factors, and prevent the adverse effects of obesity and metabolism on patients with cancer and COVID-19.

## Conclusion

Taken together, we observed that metabolic disorders, not obesity, were independently associated with higher readmission risk in patients with MM. Moreover, as the number of metabolic risk factors increased, the risk of MM readmission elevated. In the management of MM, attention should be paid to advance directives and optimized nursing for patients with metabolic disorders to reduce the readmission rate and hospitalization burden.

## Data availability statement

The raw data supporting the conclusions of this article will be made available by the authors, without undue reservation.

## Ethics statement

The studies involving human participants were reviewed and approved by Biomedical Research Ethic Committee of Shandong Provincial Hospital. Written informed consent for participation was not required for this study in accordance with the national legislation and the institutional requirements.

## Author contributions

We kindly acknowledge the participants and researchers who participated in this study. YZ, XF, KL and YFZ contributed to the design of the study protocol. YZ performed the data analyses and wrote the first draft of the manuscript. JH and ZSY contributed to the analysis plan. Chunhui Zhao contributed to the data analysis. ZNY, YC and YW revised the manuscript to create the final version. All authors proofread the manuscript and agreed with the submission and publication.
