# Supplementary material for: Association between metabolic obesity phenotypes and multiple myeloma hospitalization burden: A national retrospective study
Source: Front Oncol. 2023 Feb 23;13:1116307. doi: 10.3389/fonc.2023.1116307 (PMC9996033; doi:10.3389/fonc.2023.1116307)
Supplement: Supplementary file 1 [file Table_1.pdf]

Supplementary Table 1. Disease diagnosis codes

Diagnoses	ICD-10-CM Codes
Multiple Myeloma	C9000, C9010, C9020, C9030, C9001, C9011, C9021, C9031, C9002, C9012, C9022, C9032
Low body weight	Z681
Normal body weight	Z6820, Z6821, Z6822, Z6823, Z6824
Overweight	Z6825, Z6826, Z6827, Z6828, Z6829, E663, DE660A, E669O
Obesity of grade 1	Z6830, Z6831, Z6832, Z6833, Z6834, DE660B
Obesity of grade 2	Z6835, Z6836, Z6837, Z6838, Z6839, DE660C
Obesity of grade 3	Z6841, Z6842, Z6843, Z6844, Z6845, E6601, E662, DE660E, DE660F, DE660G, DE660H
Dyslipidemia	E780, E7800, E7801, E781, E782, E784, E7849, E785, E783, E7841
Hypertension	H35031, H35032, H35033, H35039, G932, I10, I110, I119, I120, I129, I130, I1310, I1311, I132, I150, I151, I152, I158, I159, I160, I161, I169, I674, R030, I973
Hyperglycemia	R7301, R7302, R7303, E108, E109, E1010, E1011, E1021, E1022, E1029, E10311, E10319, E10321, E103213, E103212, E103211, E103219, E10329, E103293, E103292, E103291, E103299, E10331, E103313, E103312, E103311, E103319, E10339, E103393, E103392, E103391, E103399, E10341, E103413, E103412, E103411, E103419, E10349, E103493, E103492, E103491, E103499, E10351, E103513, E103512, E103511, E103519, E103523, E103522, E103521, E103529, E103533, E103532, E103531, E103539, E103543, E103542, E103541, E103549, E103553, E103552, E103551, E103559, E10359, E103593, E103592, E103591, E103599, E1036, E1039, E1037X3, E1037X2, E1037X1, E1037X9, E1040, E1041, E1042, E1044, E1043, E1049, E1051, E1052, E1059, E10610, E10618, E10620, E10621, E10622, E10628, E10630, E10638, E10641, E10649, E1065, E1069, E1100, E1101, E1111, E1110, E1122, E1121, E1129, E11311, E11319, E11321, E113213, E113212, E113211, E113219, E11329, E113293, E113292, E113291, E113299, E11331, E113313, E113312, E113311, E113319, E11339, E113393, E113392, E113391, E113399, E11341, E113413, E113412, E113411, E113419, E11349, E113493, E113492, E113491, E113499, E11351, E113513, E113512, E113511, E113519, E113523, E113522, E113521, E113529, E113533, E113532, E113531, E113539, E113543, E113542, E113541, E113549, E113553, E113552, E113551, E113559, E11359, E113593, E113592, E113591,

	E113599, E1136, E1139, E1144, E1143, E1141, E1140, E1142, E1149, E1152, E1151, E1159, E11620, E11621, E11622, E11628, E11610, E11618, E11630, E11638, E1137X3, E1137X2, E1137X1, E1137X9, E11641, E11649, E1165, E1169, E118, E119, E0800, E0801, E0810, E0811, E0821, E0829, E08311, E08319, E08321, E083211, E083212, E083213, E083219, E08329, E083291, E083292, E083293, E083299, E08331, E083311, E083312, E083313, E083319, E08339, E083391, E083392, E083393, E083399, E08341, E083411, E083412, E083413, E083419, E08349, E083491, E083492, E083493, E083499, E08351, E083511, E083512, E083513, E083519, E083521, E083522, E083523, E083529, E083531, E083532, E083543, E083549, E083551, E083552, E083553, E083559, E08359, E083591, E083592, E083593, E083599, E0836, E0837X1, E0837X2, E0837X3, E0837X9, E0839, E0840, E0841, E0842, E0843, E0844, E0849, E0851, E0852, E0859, E08610, E08618, E08620, E08621, E08622, E08628, E08630, E08638, E08641, E08649, E0865, E0869, E088, E089, E1300, E1301, E1310, E1311, E1321, E1322, E1329, E13311, E13319, E13321, E133213, E133212, E133211, E133219, E13329, E133293, E133292, E133291, E133299,E13331, E133313, E133312, E133311, E133319, E13339, E133393, E133392, E133391, E133399, E13341, E133413, E133412, E133411, E133419, E13349, E133493, E133492, E133491, E133499, E13351, E133513, E133512, E133511, E133519, E133523, E133522, E133521, E133529, E133533, E133532, E133531, E133539, E133543, E133542, E133541, E133549, E133553, E133552, E133551, E133559, E13359, E133593, E133592, E133591, E133599, E1340, E1341, E1342, E1343, E1344, E1349, E1336, E1339, E1351, E1352, E1359, E13610, E13618, E13620, E13621, E13622, E13630, E13638, E13641, E13649, E1365, E1369, E1337X3, E1337X2, E1337X1, E1337X9, E138, E139
Heart failure	I110, I130, I132, I0981, I099, I255, I420, I425, I426, I427, I428, I429, I43, I5041, I5031, I5021, I5042, I5032, I5022, I5043, I5033, I5023, I5041, I5031, I5043, I5042, I5032, I5040, I5030, I5041, I5043, I5023, I5021, I5042, I5022, I5020
Renal failure	N171, N172, N170, N179, N178, N19, N990, R34, R944, I120, I1310, I1311, N181, N182, N183, N1830, N1831, N1832, N184, N185, N189, N186, N19, N250, Z4901, Z4902, Z940
Coronary heart disease	I249, I2510, I25110, I25111, I25118, I25119, I252
Neoplastic anemia	D630

Neutropenia	D703,D704,D708,D709
Depression	F320, F321, F322, F323, F324, F325, F328, F3281, F3289, F329, F330, F331, F332, F333, F3340, F3341, F3342, F338, F339
Stem cells transplant status	Z9484
Antineoplastic chemotherapy	Z5111
Pregnancy	Z331, Z332, Z333, Z3400, Z3401, Z3402, Z3403, Z3480, Z3481, Z3482, Z3483, Z3491, Z3492, Z3493, Z36, Z360, Z361, Z362, Z363, Z364, Z365, Z3681, Z3682, Z3683, Z3684, Z3685, Z3686, Z3687, Z3688, Z3689, Z368A, Z369, Z3A00, Z3A01, Z3A08, Z3A09, Z3A10, Z3A11, Z3A12, Z3A13, Z3A14, Z3A15, Z3A16, Z3A17, Z3A19, Z3A18, Z3A20, Z3A21, Z3A22, Z3A23, Z3A24, Z3A25, Z3A26, Z3A27, Z3A28, Z3A29, Z3A30, Z3A31, Z3A32, Z3A33, Z3A34, Z3A35, Z3A36, Z3A37, Z3A38, Z3A39, Z3A40, Z3A41, Z3A42, Z3A49, O000, O0000, O0001

Abbreviation: ICD-10-CM, Diseases-Tenth Revision-Clinical Modification.
