# Supplementary material for: Association between metabolic obesity phenotypes and multiple myeloma hospitalization burden: A national retrospective study
Source: Front Oncol. 2023 Feb 23;13:1116307. doi: 10.3389/fonc.2023.1116307 (PMC9996033; doi:10.3389/fonc.2023.1116307)
Supplement: Supplementary file 2 [file Table_2.pdf]

Supplementary Table 2. Readmission risk among different metabolic obesity phenotypes in four follow-up days

	Total Number	Number of Readmissions	HR (95% CI)	P value	aHR* (95% CI)	P value
**30-day**						
MHNO	17305	2653 (15.3%)	Reference		Reference	
MUNO	12829	2006 (15.6%)	1.019 (0.961-1.080)	0.528	1.001 (0.943-1.062)	0.981
MHO	2130	336 (15.8%)	1.031 (0.920-1.155)	0.601	1.019 (0.909-1.142)	0.751
MUO	2588	405 (15.6%)	1.020 (0.919-1.132)	0.711	0.989 (0.891-1.099)	0.841
**60-day**						
MHNO	16112	3478 (21.6%)	Reference		Reference	
MUNO	11864	2754 (23.2%)	1.079 (1.026-1.134)	0.003	1.052 (1.000-1.108)	0.052
MHO	1975	456 (23.1%)	1.075 (0.975-1.186)	0.144	1.078 (0.977-1.189)	0.135
MUO	2383	567 (23.8%)	1.109 (1.015-1.212)	0.022	1.071 (0.979-1.171)	0.134
**90-day**						
MHNO	14810	3804 (25.7%)	Reference		Reference	
MUNO	10930	3098 (28.3%)	1.113 (1.061-1.167)	< 0.001	1.074 (1.023-1.128)	0.004
MHO	1829	497 (27.2%)	1.063 (0.968-1.168)	0.198	1.068 (0.972-1.174)	0.170
MUO	2178	626 (28.7%)	1.131 (1.039-1.231)	0.004	1.089 (1.000-1.186)	0.049
**180-day**						
MHNO	10994	3702 (33.7%)	Reference		Reference	
MUNO	8107	3045 (37.6%)	1.132 (1.079-1.188)	< 0.001	1.092 (1.039-1.147)	< 0.001
MHO	1333	476 (35.7%)	1.072 (0.974-1.179)	0.153	1.087 (0.987-1.196)	0.090
MUO	1598	616 (38.5%)	1.171 (1.075-1.275)	< 0.001	1.133 (1.040-1.234)	0.004

Abbreviation: aHR, adjusted hazard ratio; CI, confidence interval; HR, hazard ratio; MHNO, metabolically healthy nonobese; MUNO, metabolically unhealthy nonobese; MHO, metabolically healthy obese; MUO, metabolically unhealthy obese.

*Adjusted COX regression: adjusted for age, sex, elective versus non-elective admission, primary payer, disposition of patient, resident,

length of stay, total charges, emergency record, same day events, patient location, antineoplastic chemotherapy, stem cells transplant status.
