# Supplementary material for: Association between metabolic obesity phenotypes and multiple myeloma hospitalization burden: A national retrospective study
Source: Front Oncol. 2023 Feb 23;13:1116307. doi: 10.3389/fonc.2023.1116307 (PMC9996033; doi:10.3389/fonc.2023.1116307)
Supplement: Supplementary file 3 [file Table_3.pdf]

Supplementary Table 3. 90-day readmission risk among different obesity metabolic phenotypes - Age stratification

	Total Number	Number of Readmissions	HR (95% CI)	P value	aHR* (95% CI)	P value
**< 65 years**						
MHNO	5483	1360 (24.8%)	Reference		Reference	
MUNO	2030	570 (28.1%)	1.149 (1.042-1.267)	0.005	1.092(0.989-1.205)	0.082
MHO	911	249 (27.3%)	1.109 (0.969-1.269)	0.134	1.101(0.961-1.261)	0.167
MUO	739	206 (27.9%)	1.125 (0.972-1.303)	0.114	1.039(0.896-1.205)	0.613
**≥ 65 years**						
MHNO	9327	2444 (26.2%)	Reference		Reference	
MUNO	8900	2528 (28.4%)	1.092 (1.033-1.154)	0.002	1.063(1.005-1.125)	0.032
MHO	918	248 (27.0%)	1.033 (0.907-1.178)	0.622	1.052(0.923-1.2)	0.445
MUO	1439	420 (29.2%)	1.132 (1.020-1.255)	0.019	1.12(1.009-1.242)	0.033

Abbreviation: HR, hazard ratio; aHR, adjusted hazard ratio; CI, confidence interval; MHNO, metabolically healthy nonobese; MUNO, metabolically unhealthy nonobese; MHO, metabolically healthy obese; MUO, metabolically unhealthy obese.

*Adjusted COX regression: adjusted for age, sex, elective versus non-elective admission, primary payer, disposition of patient, resident, length of stay, total charges, emergency record, same day events, patient location, antineoplastic chemotherapy, stem cells transplant status.
